# Pervasive translation of small open reading frames in plant long non-coding RNAs

**DOI:** 10.3389/fpls.2022.975938

**Published:** 2022-10-24

**Authors:** K. Bharathan Sruthi, Athira Menon, Akash P, Eppurath Vasudevan Soniya

**Affiliations:** Transdisciplinary Biology Lab, Rajiv Gandhi Centre for Biotechnology, Thiruvananthapuram, Kerala, India

**Keywords:** non-coding RNAs, long non-coding RNA, micro peptides, small ORF, miPEPs

## Abstract

Long non-coding RNAs (lncRNAs) are primarily recognized as non-coding transcripts longer than 200 nucleotides with low coding potential and are present in both eukaryotes and prokaryotes. Recent findings reveal that lncRNAs can code for micropeptides in various species. Micropeptides are generated from small open reading frames (smORFs) and have been discovered frequently in short mRNAs and non-coding RNAs, such as lncRNAs, circular RNAs, and pri-miRNAs. The most accepted definition of a smORF is an ORF containing fewer than 100 codons, and ribosome profiling and mass spectrometry are the most prevalent experimental techniques used to identify them. Although the majority of micropeptides perform critical roles throughout plant developmental processes and stress conditions, only a handful of their functions have been verified to date. Even though more research is being directed toward identifying micropeptides, there is still a dearth of information regarding these peptides in plants. This review outlines the lncRNA-encoded peptides, the evolutionary roles of such peptides in plants, and the techniques used to identify them. It also describes the functions of the pri-miRNA and circRNA-encoded peptides that have been identified in plants.

## Introduction

Decades ago, the notion of the C-value paradox puzzled the realm of science due to the contradiction between the genome size of eukaryotes and their complexity (Eddy, 2012). The extra non-coding DNA was considered as evolutionary remains of the genome and termed “junk” (Kuska, 1998). The advent of transcriptome sequencing revealed that most eukaryotic genomes are transcribed into non-coding RNAs (ENCODE Project Consortium, 2007). Since then, different classes of non-coding RNAs have been identified, and numerous studies have shed light on their diverse regulatory roles (Hughes et al., 2005; Tripathi et al., 2017; Lambert et al., 2019; Zhang et al., 2020). The non-coding RNAs are classified into housekeeping (tRNA and rRNA) and regulatory RNAs. Regulatory RNAs can be further divided into small non-coding RNAs (miRNA, siRNA, piRNA, Y-RNA), which possess a length of less than 200 nucleotides, and long non-coding RNAs (lncRNAs), which are longer than 200 nucleotides (Eddy, 2001; Suzuki et al., 2006; Cech & Steitz, 2014; Kowalski & Krude, 2015). Among these, lncRNAs are of particular interest owing to their low species conservation, complexity in modes of action, and presence in prokaryotes, eukaryotes, and viruses (Ma et al., 2013; Li & Yang, 2017; Wang et al., 2017; Harris & Breaker, 2018).

LncRNAs are the most prevalent non-coding RNAs that are not confined to any particular genomic region and are broadly classified into linear and circular lncRNAs based on their structure (Quinn & Chang, 2016). Linear lncRNAs can originate from intergenic, intronic, exonic, promoter, enhancer regions, and the opposite strand of coding genes (Ariel et al., 2015). Circular RNAs (circRNAs), as the name suggests, are long non-coding RNAs that have a covalently closed form and are generated mainly by back splicing events (Cocquerelle et al., 1993). Analogous to linear lncRNAs, circRNAs are also generated from intergenic, intragenic, intronic, and exonic regions within the gene (Haddad & Lorenzen, 2019). Unlike their small RNA counterparts, lncRNAs can perform different functions within the cell. LncRNAs can act in cis-mode by interacting with a nearby locus; or in trans-mode by interacting with a distant gene (Kim & Sung, 2012; Yao et al., 2019). LncRNAs are also well known for their ability to regulate the transcriptional repression of coding genes (Heo & Sung, 2011; Sanchita et al., 2020). Also, the decoying activity of lncRNA has been observed whereby it mimics the mRNAs and sequesters the miRNA during developmental and stress conditions (Franco-Zorilla et al., 2007). LncRNAs with crucial roles in stress responses and developmental processes have been identified in plants (Csorba et al., 2014; Kwenda et al., 2016; Kim & Sung, 2017; Ayachit et al., 2019; Datta & Paul, 2019; Yu & Zhu, 2019; Zhang et al., 2022). An additional function for lncRNAs as a catalyst for *de novo* gene origination has been observed in many organisms, and translatable smORFs in lncRNAs point to their coding function (Cai et al., 2008; Xie et al., 2012; Reinhardt et al., 2013).

Non-coding RNAs, in general, were thought to be devoid of any coding potential. However, recent studies have revealed that not only mRNAs but also long non-coding RNAs like lncRNAs, primary miRNAs (pri-miRNAs), and circRNAs can code for functional micropeptides, which adds to their existing complexity (Pan et al., 2018). Such pervasively translated peptides encoded by the small ORFs (smORFs) within non-coding RNAs are considered an emerging source of gene regulators in both animals and plants. Recently, the coding capacity of circRNAs has been unravelled (Chekulaeva & Rajewsky, 2019; Sinha et al., 2022). Instead of the canonical cap-dependent translation, circRNAs utilize IRESs and m6A RNA modification for coding micropeptides(P. Zhang et al., 2020). Accumulating evidence has unveiled the evolutionary role of non-coding RNAs in generating *de novo* genes. Translation of smORFs in non-coding RNAs is reported in numerous organisms using the help of ribosome profiles and proteomic data (Xie et al., 2012; Ruiz-Orera et al., 2014; Matsumoto et al., 2016; Mat-Sharani & Firdaus-Raih, 2019; Ruiz-Orera & Albà, 2019; Wu et al., 2022).

This review aims to address the emerging roles of smORFs and micropeptides encoded by linear, circular, and miRNA precursor RNAs in plants. The evolutionary role of smORFs in non-coding RNAs is briefly discussed, with an overview of various methods used for their identification.

## Small open reading frame encoded peptides

Until recently, the distinction between coding and non-coding RNAs was explicit. Numerous smORFs that encode micropeptides have been discovered in mRNAs and non-coding RNAs since the advent of ribosome profiling and bioinformatics (Hanada et al., 2007; Lin et al., 2020; Kute et al., 2021). Usually, such smORFs contain a stretch of sequences beginning with a start codon and ending with a stop codon, and they differ from conventional ORFs in terms of size (Tavormina et al., 2015). The majority of smORFs range between 2 and 100 codons in length and can be found on 5’ leader sequences, 3’ trailer sequences, the coding sequence of mRNAs or within non-coding RNAs (Chugunova et al., 2017). Based on their origin, smORFs can be classified into distinct categories like intergenic ORFs, upstream ORFs (uORFs), 3’UTR ORFs, lncRNA ORFs (lncORFs), circular RNA ORFs, and pri-miRNA coded ORFs (Lanz et al., 1999; Röhrig et al., 2002). Intergenic ORFs have been identified as the most predominant smORF category in many species. In Arabidopsis, around 3241 intergenic smORFs were identified that exhibited transcription (Hanada et al., 2007). Upstream ORFs are the second most common type of ORF generated from the upstream region. It is known that they are transcribed from the 5’ UTR of mRNAs and regulate the translation of the downstream ORFs. The translation is rarely observed in the 3’UTR region, but it has been reported in a few cells (Chugunova et al., 2017; Couso & Patraquim, 2017).

Recent studies have identified smORF encoded functional peptides in many organisms (Lanz et al., 1999; Röhrig et al., 2002). The first smORF encoded peptide identified was a 10 amino acid long peptide translated from the ENOD40 transcript in soybean (Charon et al., 1997). Previously, various smORF-encoding micropeptides in different legume species were analyzed. In total, 13 smORFs were identified from *P.vulgaris*, which had a significant role in nitrogen fixation during nodulation processes. The translated peptides from lncORFs are functional in many plants, but their conservation is less compared to the ORFs in protein-coding genes (Lin et al., 2020). It was also identified that the smORFs identified in *L.japonicus* and *M.truncatula* were unique and showed less conservation. However, most of the ORFs in *P.vulgaris* and *G.max* had orthologs in other legumes and non-legumes (Guillén et al., 2013). Another study pointed out the species-specific role of smORFs. smORFs, which showed conservation, had similarity to annotated proteins. In *P.patens*, numerous smORFs were detected that overlapped with the coding sequence of genes (Fesenko et al., 2021b). This shows that a small proportion of the identified lncRNAs are potential pseudogenes. Overexpression and knockout of *P.patens* lncORFs Pp3c9_sORF1554, Pp3c25_sORF1253, Pp3c25_sORF1000, and Pp3c18_ resulted in morphological changes (Mamaeva et al., 2022).

Circular RNAs have been identified with micropeptide coding properties in many organisms (Burd et al., 2010; Zhao et al., 2019). However, smORFs in plant circRNAs are not explored in detail. Pri-miRNAs are also regarded as a subtype of lncRNA (Morozov et al., 2021). In many plants, lncRNAs are identified to act as miRNA precursors (Sanchita et al., 2020). Similar to lncRNAs, pri-miRNAs lack long ORFs and have recently been shown to encode numerous plant micropeptides (Lauressergues et al., 2022). Two smORFs encoding 20 amino acid and 5 amino acid peptides were identified in the pri-miR171b of *M.truncatula* (Lauressergues et al., 2015). At least one putative smORF was found in the 5’end of the 50 different pri-miRNAs analyzed in Arabidopsis (Lauressergues et al., 2015).

## Functional micropeptides derived from lncRNAs and short transcripts in plants

**Figure 1:**
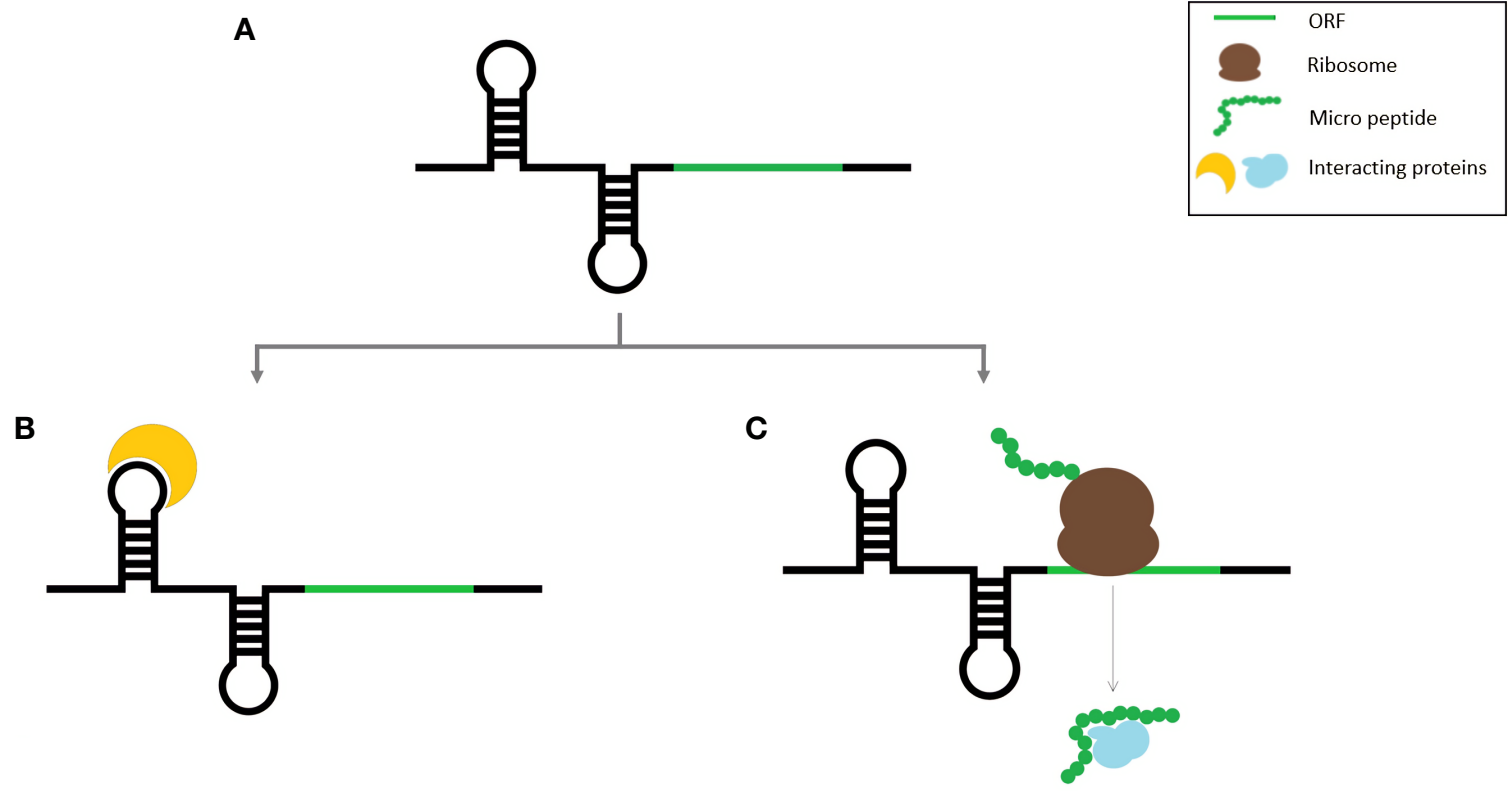
Schematic representation of bifunctional RNA **(A)** Bifunctional RNA, **(B)** RNA molecule binding to its target protein, **(C)** Micro peptide translated from the smORF within the same RNA transcript.

A striking overlap exists between the characteristics of some of the coding transcripts and lncRNAs because some lncRNAs contain one or more ORFs with coding potential. Due to the frequency with which such smORFs occur by chance, it is not unusual to find smORFs on non-coding RNAs (Tavormina et al., 2015). Transcription of intergenic regions results in the expression of a variety of transcripts, the majority of which are assumed to be long non-coding RNAs. So, there is a huge chance that many of them are protein coding. While initially a huge number of intergenic smORFs were identified in Arabidopsis, after the application of additional filters, their number was drastically reduced (Hanada et al., 2007). Identifying lncRNA-encoded peptides has other limitations as well, because often lncRNA expression is regulated temporally and spatially, which can prevent the detection of micropeptides encoded by such lncORFs. Alternatively, some non-coding RNAs have dual functions as both regulatory RNAs and micropeptides (**Figure 1**). Such RNA molecules can perform two distinct functions either in the same species or different species and are thus denoted as bifunctional RNAs (Ulveling et al., 2011; Choi et al., 2019). This can cause more ambiguity in the identification of lncRNA-encoded peptides identification.

### ENOD40 encoded micropeptide that regulates nodule development and auxin response in leguminous species

**Figure 2:**
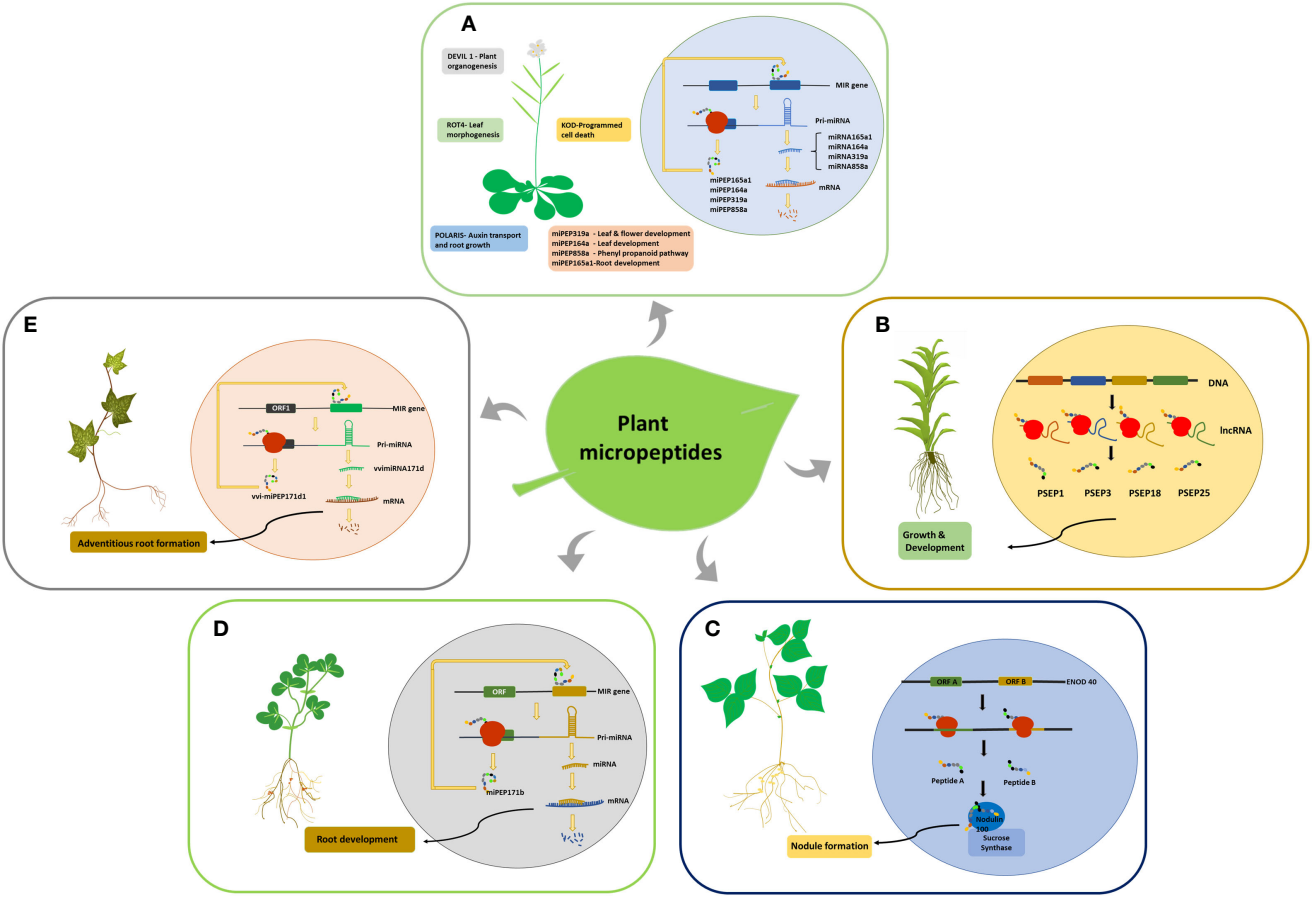
smORF encoded micropeptides identified in plants. **(A)** smORF-encoded peptides in Arabidopsis and their functions. The miPEPs function by increasing the transcription of their corresponding pri-miRNAs. **(B)** Generation of PSEPs in *Physcomitrella patens* from lncRNAs. **(C)** smORF-encoded peptides in Soybean. Peptides A and B bind to the Nodulin100 subunit of Sucrose synthase and influence nodule formation. **(D)** Generation of miPEPE171b in *Medicago truncatula* and its role in root development by increasing the transcription of its pri-miRNA. **(E)** Generation of vvi-miPEP171d1 in *Vitis vinifera* and its role in adventitious root formation by increasing the transcription of its pre-mRNA.

ENOD40 is the first micropeptide discovered in plants; it regulates symbiotic relationships between bacteria and legumes during nodule formation (Yang et al., 1993). ENOD40 codes for two short peptides of lengths of 12 and 24 amino acids, which are found in both legumes and non-legumes (Gultyaev and Roussis, 2007). The ENOD40 transcript comprises two short conserved regions, region 1 and region 2, and is devoid of a long conserved ORF, indicating that it functions primarily as an RNA (Yang et al., 1993; Röhrig et al., 2002). However, region 1 contains two small overlapping ORFs that are conserved in all ENOD40 identified leguminous species (Compaan et al., 2001). The two smORF encoded micropeptides in soybean bind to nodulin100, a subunit of sucrose synthase, and are involved in sucrose utilization during nitrogen fixation (**Figure 2** **C**). ENOD40 was also expressed at low levels in other plant organs. Multiple homologs of this RNA have been identified in monocots such as rice and maize, indicating its conserved biological function (Röhrig et al., 2002). *Medicago truncatula* and *Medicago sativa* share homology with soybean ENOD40, but neither species encodes micropeptides. Crespi et al. (1994) also found that the secondary structure of ENOD40 is more conserved than its peptides, suggesting that ENOD40 functions invariably through its RNA structure, whereas its peptide coding ability is only conserved in leguminous plants. Since both the transcripts and peptides are functional, ENOD40 can be considered a bifunctional RNA.

### Role of *Physcomitrella patens* PSEPs in growth and development

Around 70,000 transcribed smORFs were analyzed in *Physcomitrella patens* (moss), of which 5000 were conserved in multiple species. Many smORFs within mRNAs and lncRNAs were found to code for peptides. Overexpression and knockdown studies of the four selected lncRNA-encoded peptides showed morphological variations, indicating their role in moss growth and development (**Figure 2** **B**). Overexpression of PSEP1, a 41amino acid peptide encoded by lncRNA-smORF Pp3c9_smORF1544, resulted in increased filament length compared to the wild-type and knockout. The knockouts of both PSEP3, a 57 amino acid peptide, and PSEP25, a 61 amino acid long peptide, caused decreased growth and altered branching patterns, while the overexpression of PSEP25 caused only a slight decrease in growth compared to the wild type. Knocking out PSEP18, a 40 amino acid micro peptide, showed only a slight decrease in plant diameter, while its overexpression showed a significant decrease in plant diameter (Fesenko et al., 2019).

### Micropeptides encoded by lncRNAs in the root tissues of *Glycine max* and *Glycine sojae*

Recently, LC-MS/MS analysis of *G.max* and *G.sojae* root tissues revealed the presence of 153 micropeptides encoded by 179 lncRNAs. Through co-expression analysis of the protein-coding genes and micropeptides, the function of the identified micropeptides was predicted. It was observed that the protein coding genes involved in the generation of precursors of metabolites and energy, photosynthesis, light reaction, ATP synthesis coupled electron transport and regulation of defence genes were enriched. This reveals the role of the identified micropeptides in the above processes (Lin et al., 2020).

### Predicted role of maize NCPs in phenotype variation and domestication selection

The majority of the maize genome consists of functional non-coding regions, which is supported by the QTL analysis. In a recent study, a total of 1708 intergenic, 139 intronic, 89 out of frame exonic, 25 3’UTR, 18 5’UTR, and 14 from junctions, non-conventional peptides (NCPs) were identified from non-coding regions in maize. Around 70% of the micropeptides are derived from non-coding regions. The average length of identified micropeptides derived from intergenic and out of frame exonic regions was found to be greater. Also, the NCPs were enriched in the QTL regions corresponding to disease resistance, kernel length, amino acid, and oil content, which suggests their probable role in these functions. More characterization studies are required to decipher their exact role in maize (Wang et al., 2020).

### Arabidopsis POLARIS influencing root growth and phytohormone responses

A 36 amino acid coding peptide, POLARIS (PLS), was found in Arabidopsis within an auxin-inducible short transcript. The PLS transcript codes for an RNA of approximately 500 nucleotides in length (Chilley et al., 2006). Mutation of the PLS transcript causes a decrease in root length and changes in leaf vascular patterns. Exogenous cytokinin and auxin application resulted in altered responses, which indicates that the micropeptide encoded by PLS is essential for normal vascular development, root growth, and auxin and cytokinin responses (Casson et al., 2002).

### ROTUNDIFOLIA regulating leaf and flower morphogenesis in Arabidopsis

ROTUNDIFOLIA is a 53 amino acid long micropeptide with a conserved RTF domain encoded by the ROT4 ORF in Arabidopsis. The ROT4 ORF is a member of the seed plant-specific family of micropeptides which shares the 29 amino acid conserved RTF domain. Overexpression of ROT4 resulted in short leaves and floral organs, indicating its role in leaf and flower morphogenesis (Narita et al., 2004).

### Role of kiss of death in programmed cell death in plants

Programmed cell death (PCD) is a major defense system in plants against the biotic and abiotic stress. The 25 amino acid long peptide, the kiss of death (KOD), activates the PCD pathway in Arabidopsis. Mutants of this peptide showed reduced PCD in suspensor cells and root hairs under heat shock at 55°C (Blanvillain et al., 2011).

### Role of DEVIL1 in plant development

The Arabidopsis DEVIL1 (DVL1) possesses a 153-nucleotide long ORF encoding a 51 amino acid polypeptide. DVL1 overexpression resulted in phenotypic alterations, including rounder morphology of leaves, clustering of inflorescence, and a horned appearance of fruit tips. Also, DVL1 did not exhibit any similarity with known proteins. DVL1 overexpression also resulted in the downregulation of FRUITFUL, a gene involved in fruit development. This suggests DVL1 is involved in developing multiple plant organs (Wen et al., 2004).

## Circular RNA encoded micropeptides in plants

CircRNAs are covalently closed structures that are generated through back splicing and linked by their 5’ and 3’ ends. Due to the tethering of 5’ and 3’ ends with a covalent bond, circRNAs are not degraded easily by the ribonucleases (Suzuki et al., 2006). Research in circRNA is gaining momentum as they are being identified in almost all organisms. (Zhao et al., 2019; Zhang et al., 2020). Most of the identified circRNAs in animals act as sponges for miRNAs (Hansen et al., 2013). Compared to animals, the miRNA sponging activity of circRNAs in plants is considerably less (Ye et al., 2015). However, compared to animals, the characterization and functional validation of circRNA encoded peptides in plants has yet to be explored. Numerous circRNAs have been identified in both plants and animals. For example, Arabidopsis SEPALLATA3 (SEP3) derived circRNA, CircSEP3, has a role in regulating the transcription and splicing of SEP3 itself. Due to the absence of a 5’ cap and a 3’poly A tail, it was assumed that circRNAs were non-translatable. However, recently, research has revealed that their translation is possible through internal ribosome entry sites (IRES), and several ORFs have been identified in such cases. In humans, circ-FBXW7 codes for a 21 kDa peptide called FBXW7-185aa and is identified as a biomarker for glioblastoma (Yang et al., 2018). Large ORFs and m6A-modification within circRNAs are identified to encode short peptides (Zhang et al., 2020).

In maize, around 1199 circRNAs were identified, and 229 were predicted to have high coding potential. However, no autonomous peptide-coding circRNAs have been identified in plants yet. It is assumed that circRNA encoded peptides can have functions similar to those encoded by uORFs. Further studies on the molecular function of the identified circRNA encoded maize peptides need to be conducted (Han et al., 2020).

For decades, viroids have been used as the exogenous plant pathogenic circRNAs to study RNA structure and functional relationships. In one such study, the Hop stunt viroid(HSV) and Eggplant latent viroid (ELV) were used to explore their potential for coding peptides in plants. The HSV and ELV circRNA were associated with polysomes, indicating their ability to be translated. Putative ORFs with coding potential and subcellular localization signals were present in these viroids. Two HSVd ORFs, H-ORF1 (48 amino acid) and H-ORF2 (98amino acid), were identified. Three EVLd ORFs, namely E-ORF1, E-ORF2, and E-ORF3, were 110, 87, and 59 amino acids in length. None of the encoded peptides had significant similarities with any of the putative peptides. Mutations in the ORFs showed a decrease in the subcellular localization of the encoded peptides (Marquez-molins et al., 2021).

## Pri-miRNA encoded micropeptides in plants

smORFs in the 5’UTR region of pri-miRNAs code for micropeptides commonly referred to as miPEPs, which indicates the bifunctional role of pri-miRNAs. The first miPEPs identified in plants were miPEP17b from *M.truncatula* (**Figure 2** **D**) and miPEP165a from Arabidopsis (**Figure 2** **A**). miPEP171b and miPEP165a influence root development by increasing the transcription of their pri-miRNAs. The overexpression and exogenous application of these peptides resulted in an increased production of the mature miRNAs, miR165a and miR171b, which led to a decrease in lateral root development and growth of the main root (Lauressergues et al., 2015).

Grapevine pri-miR171d consists of 3 ORFs in the 5’upstream region, out of which the first ORF codes for a small peptide vvi-miPEP171d1, which increases the transcription of its pre-miRNA similar to miPEP165a and miPEP171b (**Figure 2** **E**). This results in increased transcription of vviMIR171d, which causes enhanced adventitious root formation in grapevines that can help in the commercial production of grapevines (Chen et al., 2020). Exogenous application of miPEP164a, miPEP165a, and miPEP319a in Arabidopsis to enhance the production of the corresponding miRNA (**Figure 2** **A**) and stimulate plant growth and development has been patented (Combier et al., 2017a). Similarly, the mycorrhizal symbiosis between plants and fungi was modulated with the exogenous application of miPEP171 b (Combier et al., 2017b). The production of anthocyanin in grape berry cells is significantly altered by the exogenous application of miPEP164c, which is derived from the pri-miRNA of miR164c in grapes. Targeted by the micropeptide miPEP164c is the transcription factor VvMYBPA1, a positive regulator of essential genes in the proanthocyanidin pathway. It functions by inhibiting the proanthocyanidin pathway, a competing pathway of the anthocyanin biosynthetic pathway (Vale et al., 2021).

Multiple miRNAs influencing nodule formation were identified in soybean, and their overexpression caused enhanced or decreased nodule formation. In particular, miR172c overexpression caused a positive effect on nodulation. miPEP172c was encoded by miR172c, and the exogenous application of the synthetic peptide resulted in an increased number of nodules. Nodulation marker genes like ENOD40-1, NIN, NSP, and Hb2 were highly expressed only in the miPEP172c treated plants (Couzigou et al., 2016).

Arabidopsis micropeptide miPEP858a is coded from the pri-miR858a and is required to regulate the phenylpropanoid pathway and plant growth. The micropeptide miPEP858a is crucial for the functioning of miR858a. It was revealed that the miPEP858a edited plants showed characteristics of the miR858a edited ones. The CRISPR edited and overexpression lines of miPEP858a showed significant changes in plant development and flavonoid levels (Sharma et al., 2019). Another Arabidopsis micro peptide, namely miPEP156a, is found to be evolutionarily conserved in the Brassicaceae family (Morozov et al., 2019). A few identified micropeptides encoded by lncRNAs, circRNAs, and pri-miRNAs in plants are tabulated in **Table 1**.

**Table 1:** List of plant non-coding RNA encoded micropeptides.

Organism	lncRNA name	Peptide sequence	Length (aa)	Function	Reference
*Glycine max*	*GmENOD40*	ORFA - MELCWLTTIHGS ORFB - MVLEEAWRERGVRGEGAHSSHSLT	12 24	Interacts with sucrose synthase and is required for plant–bacteria symbiotic interactions	(Röhrig et al., 2002)
*Nicotiana tobaccum* (protoplasts)	*NtENOD40*		10	Act as plant growth regulators	(van de Sandel et al., 1996)
*Physcomitrella patens*	lncRNA-sORF Pp3c9_sORF1544	*PSEP1*	41	Influences growth and development in moss	(Fesenko et al., 2019)
*Physcomitrella patens*	lncRNA-sORF Pp3c25_sORF1253	PSEP3	57	Influences growth and development in moss	(Fesenko et al., 2019)
*Physcomitrella patens*	lncRNA-sORF Pp3c25_sORF1000	*PSEP25*	61	Influences growth and development in moss	(Fesenko et al., 2019)
*Physcomitrella patens*	lncRNA-sORF Pp3c18_sORF57	*PSEP18*	40	Influences growth and development in moss	(Fesenko et al., 2019)
*Zea mays*	1652 NCPs 5’ UTR 3’ UTR intergenic intron	RMDAHALR ILTVNLKP QISVELPGVV EGTPKAVGHRQ		Predicted role in diseaseresistance, kernel length	(Wang et al., 2020)
*Arabidopsis thaliana*	*POLARIS*	MKPRLCFNFRRRSISPCYISISYLLVAKLFKLFKIH	36	Auxin transport and root growth	(Chilley et al., 2006)
*Arabidopsis thaliana*	*ROT4*	MAPEENGTCEPCKTFGQKCSHVVKKQRAKFYILRRCIAMLVCWHDQNHDRKDS	53	Leaf morphogenesis	(Narita et al., 2004)
*Arabidopsis thaliana*	Kiss of death (KOD)	MWWLVGLTPVELIHLCTFRERLCHL	25	Regulation of programmed cell death	(Blanvillain et al., 2011)
*Arabidopsis thaliana*	DEVIL1(DLV1)	MEMKRVMMSSAERSKEKKRSISRRLGKYMKEQKGRIYIIRRCMVMLLCSHD	51	Plant organogenesis	(Wen et al., 2004)
*Zea mays*	circRNAs	859 NCPs		_	(Wang et al., 2020)
*Zea mays*	circRNAs	229 cirRNAs with coding potential	5-50	_	(Han et al., 2020)
Hop stunt viroid (HSVd)	*ex*-circRNAs	H-ORF3	No stop codon	Interacts with plant translational machinary	(Marquez-molins et al., 2021)
Eggplant latent viroid (ELVd)	*ex*-circRNAs.	E-ORF1	110	Interacts with plant translational machinary	(Marquez-molins et al., 2021)
*Medicago truncatula*	miR171b	miPEP171b MLLHRLSKFCKIERDIVYIS	20	Root development -enhances the accumulation of its corresponding miRNA.	(Lv et al., 2016)
*Arabidopsis thaliana*	miR165a	miPEP165a MRVKLFQLRGMLSGSRIL	18	Root development -enhances the accumulation of its corresponding miRNA.	(Lv et al., 2016)
*Arabidopsis thaliana*	miR164a	miPEP164a MPSWHGMWLLPYWKHTHASTHTHTHNIYGC ACELVFH	37	Leaf development	(Lv et al., 2016)
*Arabidopsis thaliana*	miR319a	miPEP319a MNIHTYHHLLFPSLVFHOSSDVPNALSLHIHTYEYIIWWIDPFRITLAFR	50	Leaf and flower development	(Lv et al., 2016)
*Arabidopsis thaliana*	miR858a	miPEP858a MGGIESLLFTIVRDIGRYGTVCVVYNIKCVYTTRTKASTRTSHP	44	Phenylpropanoid pathway and development	(Sharma et al., 2019)
Soybean	miR172c	miPEP172c MWVLCLFCWPTYTHGS	16	Nodulation	(Couzigou et al., 2016)
Soybean	miR167c	miPEP167c MKGVHHFFHHKYVGLRG	17	Nodulation and lateral root development	(Couzigou et al., 2016)
*Vitis vinifera*	vvi-MIR171d 500-bp sequence upstream of premiR171d	vvi-miPEP171d1 MGYGTTPFITCKMGYGTTP	7	role in the formation of adventitious roots in grapevine	(Chen et al., 2020)
*Vitis vinifera*	miR396a	miPEP396a MLFHSFLELLF HLPN		Phenylpropanoid pathway	(Chen et al., 2020)
*Vitis vinifera*	miR164c	miPEP164c MEKQGTCITSSCTTNQ	16	Anthocyanin accumulation	(Vale et al., 2021)
*Brassica rapa*	miR156a	miPEP-156a MFCSIQCLGRHLFPLHVREIKKATKAIKKGKTL	33	miR156a represses the transition of human cancer cells from epithelium to mesenchyma Primary root formation	(Morozov et al., 2019; Erokhina et al., 2021)
*Brassica oleracea*	miR156a	miPEP-156a MFCSIQCLARHLFPLHVREIKKATKAIKKDKTL	33	_	(Morozov et al., 2019)
*Arabidopsis thaliana*	miR156a	miPEP-156a MFCSIQCVARHLFPLHVREIKKATRAIKKGKTL	33	_	(Morozov et al., 2019)
*Arabis alpine*	miR156a	miPEP-156a MFWSIQSLARHLFSLHVREIIKRQKP	26	_	(Morozov et al., 2019)
*Boechera stricta*	miR156a	miPEP-156a MVCSIQCLARHLFPLHVREIKKATKIIKKGKTL	33	_	(Morozov et al., 2019)
*Capsella bursa*	miR156a	miPEP-156a CFCSIQCLARHLFPLHVREIKKATKSHKERVRRDSLFER	39	_	(Morozov et al., 2019)
*Barbarea vulgaris*	miR156a	miPEP-156a MFCSIQCLTRHVFPFACKRDKESDKSHKER	30	_	(Morozov et al., 2019)
*Conringia planisiliqua*	miR156a	miPEP-156a MFCSIQCLARHLFPLHVREIKKATKAIKKGKTL	33	_	(Morozov et al., 2019)
*Euclidium syriacum*	miR156a	miPEP-156a WFCSIQCLARLLFPLHVREIKKATKAIKKGNTLSKVER	38	_	(Morozov et al., 2019)
*Eutrema yunnanense*	miR156a	miPEP-156a IFCSIQCLARHVFPLHVREIKKATKAIKKGKTL	33	_	(Morozov et al., 2019)
*Thlaspi arvense*	miR156a	miPEP-156a MPCQHLFPLHVREIKKATKAIKKGKTL	27	_	(Morozov et al., 2019)
*Caulanthus amplexicaulis*	miR156a	miPEP-156a MPRRHLFPLHVREIKKPTKAIKKDLWSWKNCE	32	_	(Morozov et al., 2019)

## Evolutionary significance of small ORFs

Previously, it was believed that only a minute fraction of the genome was translatable. Pervasive translation has revealed that translatable non-coding regions dominate the genome (Crappé et al., 2014; Housman & Ulitsky, 2016). Approximately 20% of the eukaryotic genome is comprised of genes with no sequence similarity to those of other species (Khalturin et al., 2009). These genes are termed orphan genes. Gene duplications, horizontal gene transfers, retro transposition, exon shuffling, and frame shift mutations are the most prevalent mechanisms through which orphan genes are generated. More recently identified mechanisms include the origin of *de novo* genes from non-coding regions such as introns, 3’ and 5’ untranslated regions, and intergenic regions (Long et al., 2003; Khalturin et al., 2009; Schlotterer, 2015). Nearly 5.5% of orphan genes identified in primates are derived from non-coding regions (Toll-Riera et al., 2009).

Recently, *de novo* gene birth from previously annotated non-coding RNAs is gaining traction and has been observed in numerous vertebrates, plants, yeast, and other species (Cai et al., 2008; Knowles and Mclysaght, 2009; Reinhardt et al., 2013; Chen et al., 2015). Unlike protein-coding genes, *de novo* genes are shorter, lack homology, and are not well conserved across species (Cai et al., 2008; Reinhardt et al., 2013; Bornberg-Bauer et al., 2015; Guerzoni and McLysaght, 2016). *De novo* genes were first identified in *Drosophila melanogaster* and *Drosophila yakuba*, and their transcriptional history revealed that they originated from lncRNAs, indicating that *de novo* proteins were initially transcribed and later acquired the ability to encode proteins (Reinhardt et al., 2013). Acquisition of an ORF and integration of the regulatory signals necessary for translation are the two essential steps in the generation of *de novo* genes. There is still a debate concerning the sequence of these two steps, and hence, there are two models: the transcript first model and the proto-ORF model (Reinhardt et al., 2013). According to the transcript first model, the majority of the genome is transcribed, and a considerable fraction of these transcripts are associated with ribosomes. In order for a non-coding RNA to translate proteins or short peptides, the non-coding sequence should first be transcribed, and then various mutations must create a translatable ORF. This model is observed in BSC4, a novel gene in *Saccharomyces cerevisiae* that evolved from a non-coding sequence (Cai et al., 2008). The proto-ORF model proposes that ORFs already exist in the transcripts and they await the acquisition of regulatory elements for the origination of novel genes (Reinhardt et al., 2013; Schlotterer, 2015). The Poldi gene in *Mus musculus* exemplifies this model. This gene emerged from an intergenic non-coding region 2.5 to 3.5 million years ago and already contained the ORFs and transcription signals (Heinen et al., 2009). Due to the accumulation of mutations, the non-coding origin of ancient proteins cannot be predicted. However, the recently evolved species-specific novel genes can be probed to identify their origin (Cai et al., 2008). Due to this, the functionality of such genes has been in question and several methods have been adopted to confirm their authenticity. Protein-coding genes are frequently subjected to purifying selection since they cannot sustain deleterious mutations. Consequently, the presence of purifying selection in *de novo* genes demonstrates their functional nature (Carvunis et al., 2013; Schlotterer, 2015). Ribosome profiling and mass spectrometry studies provide evidence for the translation of *de novo* genes into peptides or proteins (Xing et al., 2021). Knock down of *de novo* genes helps to decipher their functionality. In Drosophila, knockdown of some of the *de novo* genes resulted in a lethal phenotype (Liu et al., 2014). In a constitutive RNAi knockdown experiment in Drosophila, 59 genes were found to be lethal (Chen et al., 2010). Even though *denovo* genes are not expressed constitutively, their differential expression signifies their functional nature. An example is the lethality of *de novo* genes at various stages of development observed in Drosophila (Chen et al., 2010). A strong correlation between *de novo* genes’ transcription profiles and their transcripts is observed only when novel proteins are evolved from functional RNA transcripts. Comparative transcription profiling in humans, chimpanzees, and rhesus macaques revealed 24 hominid-specific *de novo* genes with an identical transcriptional profile (Xie et al., 2012).

Some non-coding transcripts can possess multiple ORFs, including primary ORFs, interORFs, and other ORFs. A study conducted in six different eukaryotic species showed that the ribosome binds majorly to primary ORFs and other ORFs. Also, around 30-82% of lncRNA transcripts were ribosome protected, suggesting the presence of translatable ORFs in the lncRNAs of these species. Among these, the Arabidopsis lncRNA AT1G34418.1 contains other ORFs coding for 2 and 12 amino acid long peptides along with a primary ORF (Ruiz-Orera et al., 2014).

Using *Physcomitrella patens* lncRNAs as a reference, a recent study deciphered a comprehensive analysis of the conservation of smORFs across 479 plant species. The conservation of smORFs was found to depend on their similarity to annotated or predicted proteins. About 3% of lncRNAs were discovered to be remnants of ancestral protein-coding genes. Some of the smORF-encoded peptides identified in this study were incorrectly characterised as lncRNA-encoded, as they were small functional proteins or peptide precursors. A few of the identified smORFs in this study manifested poor species conservation and, through positive selection, could be a rich source of micropeptides. In addition, some of the identified translatable smORFs in the plant species revealed only nucleotide-level conservation. This could suggest a significant role in the evolution of plant smORFs in de-novo gene birth (Fesenko et al., 2021).

## Methods used for the discovery of lncRNA encoded peptides

Multiple studies have identified that lncRNAs associate with ribosomes, indicating that they could encode peptides. With the increasing importance of such non-coding RNAs, research is being directed towards identifying the peptides encoded by these sequences, for which different methods are being employed.

### Bioinformatics analysis

The advancement of bioinformatics has led to a better understanding of lncRNAs and their roles in different organisms. It has also helped to reveal that lncRNAs have the potential to encode small but functional peptides, which was previously not known.

Bioinformatics analysis is often the first step in identifying peptides encoded by lncRNAs. Different regions of the genome are scanned for the presence of ORFs that code for small peptides. In some cases, specific lncRNAs, circRNAs, and miRNAs are chosen, and the sequences and the surrounding regions are screened for the presence of putative open reading frames. The coding potential of lncRNAs is usually predicted by scanning for the start codon, AUG/ATG, or regulatory elements like IRES and m6A sites. These markers are used for prediction as an ORF usually starts with AUG/ATG, and the regulatory elements help mediate translation. Some examples of ORF prediction tools included are ORF Finder, ORF Predictor, IRESite, IRESfinder, M6APred-EL, M6AMRFS (Ye et al., 2020). Classical gene prediction pipelines generally have an ORF cut off. As a result, they often fail to identify smORFs due to their short length. Moreover, many smORFs use non-AUG initiation codons and also lack significant sequence conservation. This lack of consensus features, makes the prediction of smORFs much more complicated as compared to gene prediction (Mat-Sharani & Firdaus-Raih, 2019).

New prediction tools have been developed that take diverse features into consideration to identify smORFs. They mainly look for conservation of the smORF among different species which would indicate that they could have a conserved biological function and are unlikely to be artefacts (Chugunova et al., 2018). CRITICA is a coding region identification tool that compares the query DNA with related DNA sequences from other species to look for amino acid conservation (Badger et al., 1999). phastCons is a program based on a phylogenetic hidden Markov model that can identify conserved elements in a multiple alignment (Siepel et al., 2005). PhyloCSF is a prediction tool that also assesses the coding potential of a transcript by comparing it with informant genomes that have already been annotated (Lin et al., 2011). micPDP is a computational pipeline that was used to identify micropeptides by analyzing codon conservation patterns in multiple species alignments of human lincRNAs and fish transcripts (Bazzini et al., 2014). uPEPperoni is an online tool that identifies open reading frames in the 5’ untranslated regions of mRNA by comparing the query sequence with sequences in the NCBI RefSeq database (Skarshewski et al., 2014).

Prediction tools are developed that analyze the codon usage, characteristic features of the coding regions and sequence similarity to previously identified proteins or functional domains (Chugunova et al., 2018). CPC is a prediction tool that uses six biologically meaningful features to assess the coding potential of a transcript (Kong et al., 2007). Lncindent is an alignment free tool which uses sequence intrinsic composition and open reading frame information (Han et al., 2016). COME utilizes both sequence features and experimental data to predict the coding potential with more accuracy and consistency (Hu et al., 2017). CNIT is a tool that uses the intrinsic sequence composition to classify protein-coding RNAs and hence can potentially be used in species without a whole genome sequence or poorly annotated information (Guo et al., 2019). MiPepid is a machine-learning tool designed to identify micropeptides from DNA sequences by analyzing the nucleotide patterns (Zhu & Gribskov, 2019). RNAmining is a standalone and web server tool that uses the XGBoost algorithm to predict the coding potential of ncRNA by mainly analyzing the trinucleotide count and sequence length (Ramos et al., 2021). There are also specific tools for identifying peptide-coding circRNAs like CircCode which is a tool based on Python 3 that identifies translated circRNAs from ribo-Seq data (Sun and Li, 2019). CircPro is also a tool that can detect cirRNAs, predict its peptide-coding potential and identify junction reads from ribo-seq data (Meng et al., 2017). Such bioinformatics tools were employed to discover peptide-encoding lncRNAs in *Physcomitrella patens* and miPEPs in *Arabidopsis*, *Brassica*, and *Vitis vinifera* (Lauressergues et al., 2015; Fesenko et al., 2019; Morozov et al., 2019; Sharma et al., 2019; Chen et al., 2020). Even though, bioinformatic analysis allow us to identify potentially peptide-encoding smORFs, they cannot be completely relied upon. smORF prediction is now being complemented with transcriptomic and proteomic data as indirect and direct evidence of translation (Hellens et al., 2016). Various databases and prediction tools for smORFs and micropeptides have been listed in **Table 2**.

**Table 2:** List of databases and prediction tools for smORFs and micropeptides.

Type	Description	URL and Running environment	Reference
**Database/Repository**			
FuncPEP	A database of functional peptides from non-coding regions of the genome	https://bioinformatics.mdanderson.org/Supplements/FuncPEP/database.html -Web server	(Dragomir et al., 2020)
SmProt	A database of small proteins encoded by annotated coding and non-coding RNA loci	http://bigdata.ibp.ac.cn/SmProt/ -Web server	(Hao et al., 2018)
PsORF	A database of small ORFs in plants	http://psorf.whu.edu.cn/#/	(Chen et al., 2020)
sORFS.ORG	A repository of small orfs identified by ribosome profiling	http://www.sorfs.org/ -Web server	(Verbruggen et al., 2016)
TransCirc	An interactive database for translatable circular RNAs based on multi-omics evidence	https://www.biosino.org/transcirc/	(Huang et al., 2021)
SPENCER	A comprehensive database for small peptides encoded by ncRNA in cancer patients	http://spencer.renlab.org/#/home -Web server	(Luo et al., 2022)
ARA-PEPs	A repository of putative sORFencoded peptides in Arabidopsis thaliana	http://www.biw.kuleuven.be/CSB/ARA-PEPs -Web server	(Hazarika et al., 2017)
ncEP	A Manually Curated Database for Experimentally Validated ncRNA-encoded Proteins or Peptides	http://www.jianglab.cn/ncEP/ -Web server	(Liu et al., 2020)
**Web tools**			
CRITICA*	Coding region identification tool invoking comparative analysis	http://rdpwww.life.uiuc.edu/	(Badger et al., 1999)
sORF finder*	Analysis of nucleotide sequence composition and conservation at the amino acid level	http://evolver.psc.riken.jp/	(Hanada et al., 2010)
CPC	A fast and accurate coding potential calculator based on sequence intrinsic features	http://cpc2.gao-lab.org/ -Web server	(Kong et al., 2007)
CNIT	A fast and accurate web tool for identifying protein-coding and long non-coding transcripts based on intrinsic sequence composition	http://cnit.noncode.org/CNIT -Web server	(Guo et al., 2019)
COME	A robust coding potential calculation tool for lncRNA identification and characterization based on multiple features	https://github.com/lulab/COME -Web server	(Hu et al., 2017)
RNAmining	A machine learning stand-alone and web server tool for RNA coding potential prediction	https://rnamining.integrativebioinformatics.me/ -Web server	(Ramos et al., 2021)
Lncident	A Tool for Rapid Identification of Long Noncoding RNAs Utilizing Sequence Intrinsic Composition and Open Reading Frame Information	http://csbl.bmb.uga.edu/mirrors/JLU/Lncident/annotate.php -Web server	(Han et al., 2016)
MiPepid	MicroPeptide identification tool using machine learning	https://github.com/MindAI/MiPepid -Web server	(Zhu & Gribskov, 2019)
PhyloCSF	A method to determine whether a multi-species nucleotide sequence alignment is likely to represent a protein-coding region	https://github.com/mlin/PhyloCSF/wiki	(Lin et al., 2011)
phastCons	Part of a software package called PHAST (PHylogenetic Analysis with Space/Time models), which is available by request from acs@soe.ucsc.edu	http://compgen.cshl.edu/phast/	(Siepel et al., 2005)
micPDP*	Quality of the ORF (ORF size, coverage, integrity) and conservation		(Bazzini et al., 2014)
uPEPperoni*	An online tool for upstream open reading frame location and analysis of transcript conservation	http://upep-scmb.biosci.uq.edu.au/	(Skarshewski et al., 2014)
CircCode	Tool for Identifying circRNA Coding Ability	https://github.com/PSSUN/CircCode	(Sun and Li, 2019)
CircPro	An integrated tool for the identification of circRNAs with protein-coding potential	http://bis.zju.edu.cn/CircPro/	(Meng et al., 2017)

*Web tool not available anymore.

### Experimental validation

Although bioinformatic analysis allows the identification of lncRNAs with the potential to code for peptides, it has to be determined whether the ORFs are translated and functional *in-planta*. Ribosome profiling (ribo-seq) has emerged as a standard method to detect peptide-coding non-coding RNAs. This technique reveals RNA sequences that associate with translating ribosomes or ribosome protected fragments (RPFs) and hence can be used to identify sequences that could code for a peptide. However, this technique does not provide direct evidence of translation. They are based on the assumption that if a ribosome is associated with a sequence, it would code for a peptide, which is considered as one of the disadvantages of this method (Zhang et al., 2021).

Nowadays initiation blockers like harringtonin or lactimidomycin are being used to halt the ribosomes in order to accurately determine the initiation site. This is particularly helpful in determining the initiation site when multiple putative smORFs are present in all three reading frames. The Poly-Ribo-Seq method, in which profiling is carried out for sequences that are associated with polysomes that represent active translation, also increases the accuracy of detection (Chugunova et al., 2018; Kute et al., 2021).

Unlike ribo-seq, mass spectrometry (MS) identifies the lncRNA-encoded peptides themselves rather than the lncRNA sequence and has been used to identify such micropeptides. Even though MS is the gold standard for detecting peptides, it is analytically challenging, and the number of micropeptides detected is less (Zhang et al., 2021). Short peptide sequences (<10aa) and the use of reference databases also limit MS from detecting lncRNA-encoded peptides and novel micropeptides (Ye et al., 2020). Fabre et al., 2021, have summarized the recent developments in MS-based peptidomics workflows specifically to identify smORF-encoded peptides. In the case of plants, MS has been used to confirm the presence of micropeptides in Arabidopsis, Soybean*Physcomitrella patens* and maize (Fesenko et al., 2019; Wang et al., 2020)

Another method used is the in-fusion expression of the β-glucuronidase (GUS) reporter gene and the identified ORFs followed by immunofluorescence to detect the presence of the peptides *in planta* (Sharma et al., 2019). Tagging a Flag or GFP fusion protein at the C-terminal end followed by immunofluorescence or western blotting to detect the presence of the tagged peptide is another commonly used method. Specific monoclonal antibodies are used to confirm the presence of the peptides (Ye et al., 2020). This method has been used in *Arabidopsis* and *Medicago truncatula*. (Lv et al., 2016; Ye et al., 2020).

Various databases contain information about lncRNA encoded peptides; however, no database is dedicated explicitly to micropeptides found in plants. sORFs.org contains peptide-coding ORFs identified through ribosome profiling, while the Smprot database contains sequences predicted using both ribosome profiling and MS (Verbruggen et al., 2016; Hao et al., 2018). The ncEP database provides a collection of low-throughput experimentally validated non-coding RNA-encoded peptides sourced from published articles (Liu et al., 2020). The FuncPEP database contains ncRNAs encoding peptides that are biologically functional. The ncRNAs in the FuncPEP database have been validated through indirect methods like ribosome profiling and loss of function techniques or *via* direct methods like MS, western blotting and immunostaining (Dragomir et al., 2020).

SPENCER is a database that contains non-coding RNA encoded small peptides from 15 different cancer types identified through MS-based proteomics (Luo et al., 2022). The PsORF database was constructed using released genomic, transcriptomic, ribo-seq and MS data of 35 different plant genomes and has made available a set of non-coding region encoded smORFs (Chen et al., 2020). An Arabidopsis specific database, the ARA-PEP database, contains smORF-encoded peptides in *A. thaliana* compiled from Tiling arrays and RNA-seq data in response to biotic and abiotic stress (Hazarika et al., 2017). TransCirc is a database that specifically contains circRNAs with peptide-encoding potential which were compiled based on both direct and indirect evidences (Huang et al., 2021).

The discovery of these micropeptides is still low due to their poor predictability, small size, and low abundance. The different methods that have been used in the past have their advantages and disadvantages (Fabre et al., 2021; Pei et al., 2022). Studies suggest that combining multiple methods could help to increase the accuracy of detection as seen in the case of *Physcomitrella patens* where the smORFs were identified using publicly available lncRNAs datasets and the MiPepid tool which were then validated using transcriptome and proteome analysis (Fesenko et al., 2021) (Ye et al., 2020).

## Conclusion and future perspectives

In the recent past, diverse roles of lncRNAs in plants and animals have been explored extensively. However, the functional elucidation of plant lncRNA, miRNA, and circRNA encoded peptides are still in its infant stage. LncRNA encoded peptides have only been identified in a few plant species and further extensive studies are needed to explore the extent of functional micropeptides and decipher their crucial role in the plant kingdom. Majorly the identified micropeptides in plants are coded by the pri-miRNA, and most function in regulating their corresponding miRNA. In the future, exogenous application of miRNA encoded peptides can allow their utilization as molecular pesticides and fertilizers, thereby reducing the adverse effects generated by using chemical equivalents. Previously, the developmental role of exogenous application of micropeptides have been researched and patented. Other possible roles of miPEPs besides feedback regulation must be explored in detail.

This review aims to traverse the coding realm of plant non-coding RNAs in general and lncRNAs in particular, with their implications in regulatory functions. The gap between identifying lncRNA encoded peptides and their functional characterization are addressed. Further, we have discussed the potential evolutionary roles of lncRNAs in the *de novo* gene birth of the protein-coding genes. Moreover, the methodology adapted to identify smORFs, and their translated peptides have been reviewed in detail.

## Author contributions

KBS and EVS conceived the idea. KBS and AM contributed to the writing of the manuscript and revision. EVS contributed to the editing and revision of the manuscript. AP assisted in preparing the figures and revision of the manuscript. All authors contributed to the article and approved the submitted version.

## Funding

This work was supported by the University Grants Commission under CSIR/UGC Fellowship and Department of Biotechnology, Government of India (DBT).

## Conflict of interest

The authors declare that the research was conducted in the absence of any commercial or financial relationships that could be construed as a potential conflict of interest.

## Publisher’s note

All claims expressed in this article are solely those of the authors and do not necessarily represent those of their affiliated organizations, or those of the publisher, the editors and the reviewers. Any product that may be evaluated in this article, or claim that may be made by its manufacturer, is not guaranteed or endorsed by the publisher.
